# Monitoring and Management of Childhood Asthma in Asian Countries *A Questionnaire Study*

**DOI:** 10.1097/WOX.0b013e318194c0f6

**Published:** 2009-01-15

**Authors:** Belle Wong, Colin Tan, Bee Wah Lee, Hugo P Van Bever, the APAPARI Study Group

**Affiliations:** 1 Department of Paediatrics, The Children's Medical Institute, National University Singapore, National University Hospital, 5 Lower Kent Ridge Rd, 119074 Singapore

**Keywords:** childhood asthma, physicians, questionnaire, Southeast Asia

## Abstract

**Background**

A recent study by the International Study of Asthma and Allergies in Childhood has shown that asthma symptom prevalence is still increasing in parts of Asia. As such, it is important to know how well asthma is being managed. Practices of physicians in India, China, Sri Lanka, Australia, Singapore, Indonesia, Philippines, and Taiwan in monitoring and treating childhood asthma were examined.

**Methods**

A 6-page standardized questionnaire was sent to physicians via post. The questionnaire is made up of 3 parts, including (1) methods of monitoring of childhood asthma, (2) practices in managing acute asthma exacerbations, and (3) choice of therapy in maintenance treatment.

**Results**

Our study reflects mostly the practices of physicians who practice in urban regions. Of respondents, 41.4% were general pediatricians, whereas 26.3% were general practitioners. A small fraction of physicians used score cards or diaries to monitor asthma, ranging from 0% (Philippines and Australia) to 15.9% (India). Only 8.1% (Sri Lanka) to 52.0% (Australia) use either a peak flow meter and/or spirometry to monitor asthma. However, for frequency of use, 35% (China) to 94% (Indonesia) never or seldom make use of a peak flow meter, and 33% (China) to 97.6% (Indonesia) never or seldom use spirometry for monitoring. Most physicians treat acute asthma appropriately with short-acting bronchodilators. For maintenance treatment, an inhaled corticosteroid was the most frequently chosen first-choice therapy. However, a significant fraction of physicians chose a long-acting β-agonist monotherapy as a first-choice treatment for asthma maintenance. For infants, the percentage ranged from 1.4% (Australia) to 76.3% (Indonesia); in preschoolers, 1.8% (Australia) to 43.3% (Indonesia); and in older children, 0% (Philippines) to 28.8% (Indonesia). These results may be related to the overall affluence of each nation.

**Conclusions**

There is much room for improvement in increasing physicians' awareness to guidelines for more effective management of pediatric asthma in Southeast Asia, especially regarding the high use of long-acting β-agonist monotherapy, even in young children.

## 

Arecent report of a worldwide collaborative survey of asthma and allergic diseases in children (International Study of Asthma and Allergies in Childhood) has shown that asthma symptom prevalence is still increasing in parts of Asia [1]. Asthma has been shown to start early in life. In Singapore, 11.7% of preschoolers aged 4 to 6 years have already been diagnosed with asthma [2]. In fact, 60% of children with asthma develop their symptoms before the age of 3 years [3]. In a cross-sectional study done in Singapore, 22.9% of children in their second year of age already had the asthma-related symptom of wheezing [4]. This indicates that there is a need for asthma intervention in early years, thus highlighting the importance of good asthma management in children. There is also evidence to suggest that severity of asthma in Asia is worsening. Increasing asthma admission rates in Thailand have been reported, demonstrating a worsening in asthma severity in Thailand during the last decade [5]. As such, our aim was to examine practices of physicians in Southeast Asia in treating childhood asthma.

## Materials and methods

The study was performed using a standardized questionnaire that was distributed among randomly selected groups of physicians. The questionnaire comprises 6 pages and 29 items on (1) demographic data of the participant; (2) monitoring of childhood asthma; (3) treating an acute asthma attack in children; and (4) maintenance treatment of childhood asthma in infants, preschoolers, and older children. The main points of the questionnaire are summarized in Table 1. Most of the countries studied had had the questionnaire distributed via post. Distribution of the questionnaires was mostly around urban regions.

**Table 1:** Questionnaire Details

Section	Subheading	Questions
A	Demographic data	Sex, age, speciality, employment
B	Monitoring childhood asthma	Symptom score cards/diary cards/peak flow meter/spirometry use
C	Treating an acute asthma attack in children	Drug of choice, criteria for admission, use of bronchodilators, use of corticosteroids, oxygen therapy, and antibiotics
D	Maintenance treatment of childhood asthma in children	Drug of choice, frequency of use of maintenance treatment, criteria to determine starting a maintenance treatment. Drugs include montelukast, inhaled corticosteroids, and long-acting bronchodilators

The areas studied were Padang and Jakarta (Indonesia), Perth (Australia), New Delhi (India), 25 cities in China, Metro Manila (Philippines), and the whole countries of Taiwan, Singapore, and Sri Lanka.

Responses were collected via reply e-mails within a set 3-month period for reply and were then tabulated by 2 investigators with cross-checking of results. This was subsequently analyzed with SPSS Version 13.0. Descriptive statistics were generated, and various cross tabulations and tables were produced as a result.

## Results

### Demographic data

In total, 1905 respondents were surveyed from the 8 countries in Southeast Asia (Table 2). Response rates varied from 8.3% in Singapore to 34.6% in Taiwan, although significantly higher response rates of 65% and 79.9% were observed in the Philippines and Indonesia, respectively. Fifty-five percent of the respondents were females, and 45% were males. Ages of most responders ranged between 31 and 45 years, the percentage being 55. A large group of the respondents were general pediatricians (41.4%), followed by general practitioners, making up the next 26.3%.

**Table 2:** Number of Respondents Per Country

	India	Australia	Sri Lanka	Taiwan	China	Indonesia	Singapore	Philippines
No. respondents	82	232	49	101	912	215	173	143

### Monitoring childhood asthma

Trends for this category were relatively similar for all the countries. Only a small fraction of physicians used score cards or diaries to monitor asthma, ranging from 0% (Philippines and Australia) to 15.9% (India). Generally, a slightly higher percentage of physicians monitor pediatric asthma using both peak flow meters and spirometry, as compared with only with peak flow meters. A small minority only use spirometry for monitoring of asthma (Table 3).

**Table 3:** Percentages of Respondents Who Monitor Asthma With Peak Flow Meters or Spirometry

	India	Australia	Sri Lanka	Taiwan	China	Indonesia	Singapore	Philippines
Percentage of respondents who *always *monitor asthma with the following								
Both peak flow and spirometry	29.3	44.2	2	25.7	29	6.5	7.5	8.4
Peak flow only	15.8	5.2	6.1	5	8	0.5	8.1	0
Spirometry only	0	2.6	0	0	5	0	0.6	0
Percentage of respondents who *seldom *or *never *monitor asthma with the following								
Peak flow meter	43.8	47.4	75.5	45.6	35	94	47.6	69.9
Spirometry	81.6	64.5	100	85.2	33	97.6	55.6	95.8

The frequency of monitoring of asthma using peak flow and spirometry was noted to be exceedingly low, as a large percentage of physicians indicated that they seldom or never use spirometry and peak flow for monitoring.

### Treating Acute Asthma Attacks in Children

Nebulized salbutamol given every 20 minutes was generally favored as the treatment of choice in treating acute asthma attacks by most physicians in each country (Table 4). In China, an additional 22% stated that they would opt for temporarily administering inhaled salbutamol or terbutaline and adopting a "wait-and-see" approach. Between one quarter and two thirds of physicians frequently or always ad-minister systemic corticosteroids in an outpatient setting (emergency department or polyclinic) in the treatment of acute asthma, whereas the overwhelming majority would use corticosteroids in the inpatient setting. The duration of choice was generally cited to be 3 to 5 days. Physicians were mixed in their use of antibiotics, and few physicians favored high-dose inhaled corticosteroids in acute management. Majority of over 80% per country never or seldom used aminophylline as a first-line treatment of asthma.

**Table 4:** Treatment of Acute Attacks

	India	Australia	Sri Lanka	Taiwan	China	Indonesia	Singapore	Philippines
Salbutamol*	68.2	41.2	85.7	51.5	29	54.9	60.7	66.4
Systemic corticosteroids**	52.4	50.3	51.0	68.3	50	30.7	41.0	27.3
Inhaled corticosteroids^‡^	48.7	49.4	26.5	39.6	82	47.9	60.1	54.6
Use of corticosteroids in admitted patients^§^	98.7	87.5	100	100	99	94.9	84.4	100
Length of prescription of corticosteroids^||^	53.6	73.2	93.9	56.4	79	77.8	63.7	68.5
Antibiotics^¶^	40.2	47.4	32.7	80.2	35	50.7	52.1	50.3
High-dose inhaled corticosteroids^#^	6.09	4.3	0	1	3	0	3	8.4

*Percentage of physicians who stated nebulized salbutamol given every 20 minutes as first choice for treatment of an acute asthma attack.

**Percentage of physicians who frequently or always administered systemic corticosteroids in an outpatient setting (emergency department/polyclinic) in the treatment of acute asthma.

^‡^Percentage of physicians who frequently or always administered inhaled corticosteroids in an outpatient setting (emergency department/polyclinic) in the treatment of acute asthma.

^§^Percentage of physicians who use corticosteroids in admitted patients.

^||^Percentage of physicians who give 3 to 5 days of corticosteroids at the same dosage throughout that period for acute asthma.

^¶^Percentage of physicians who give antibiotics for cases of acute asthma.

^#^Percentage of physicians who use high-dose inhaled corticosteroids for acute asthma.

### Maintenance treatment of childhood asthma in children

Severity and frequency of the symptoms were the 2 main criteria physicians used to determine whether to start a maintenance treatment in children with asthma. This ranged from 19.5% (India) to 46.9% (Sri Lanka) for severity and 36% (China) to 73.1% (India) for frequency.

A significant fraction of physicians chose long-acting β-agonist (LABA) monotherapy as first-choice treatment for asthma maintenance (Table 5). Inhaled corticosteroid therapy was selected as a first-choice therapy for asthma maintenance in infants by 11.0% (Indonesia) to 81.6% (Sri Lanka) of physicians. In preschoolers, it ranged from 10.0% (Indonesia) to 69.4% (Sri Lanka) of physicians, and in older children, it ranged from 24.0% (Indonesia) to 69.4% (Sri Lanka) (Table 6).

**Table 5:** Percentages of Physicians Who Use LABA Monotherapy as a First-Choice Treatment for Asthma Maintenance in the Specified Age Groups

	India	Australia	Sri Lanka	Taiwan	China*	Indonesia	Singapore	Philippines
Infants	9.8	1.4	4.1	6.9	60	76.3	8.1	7.7
Preschoolers	15.9	1.8	10.2	8.9	58	43.3	8.7	7.7
Older children	3.8	2.2	6.1	5.9	59	28.8	5.8	0

*Results for this country may include physicians who may use LABA as both monotherapy and add-on.

**Table 6:** Percentages of Physicians Who Use Inhaled Corticosteroids as a First-Choice Treatment for Asthma Maintenance in the Specified Age Groups

	India	Australia	Sri Lanka	Taiwan	China	Indonesia	Singapore	Philippines
Infants	52.4	34.5	81.6	58.0	85.0	11.0	36.4	30.8
Preschoolers	48.7	38.8	69.4	41.1	83.0	10.0	46.2	35.0
Older children	42.6	40.4	69.4	59.8	81.0	24.0	42.2	35.0

Montelukast use as a first-choice treatment was favored by 0.0% (Sri Lanka, Philippines) to 42.4% (Taiwan) of respondents in infants. For preschoolers, the range was from 1.9% (Indonesia) to 45.3% (Taiwan) of respondents, and in older children, it ranged from 0.0% (India, Sri Lanka) to 19.8% (Taiwan) and 72% (China) (Tables 6 and 7).

**Table 7:** Percentages of Physicians Who Use Montelukast as a First-Choice Treatment for Asthma Maintenance in the Specified Age Groups

	India	Australia	Sri Lanka	Taiwan	China	Indonesia	Singapore	Philippines
Infants	8.5	14.5	0.0	42.4	72.0	1.9	12.7	0.0
Preschoolers	10.9	22.5	2.0	45.3	74.0	1.9	11.0	7.7
Older children	0.0	9.2	0.0	19.8	72.0	1.4	8.1	15.4

## Discussion

There are little data on practices of asthma management in Southeast Asia, although this has been widely studied in developing and developed parts of western countries [6-8]. Judging from the recent trend of increasing pediatric asthma cases in this region,[1] and as the economic cost of asthma has been found to be considerable in populations outside the western hemisphere (this has been found to be approximately US $238 per asthmatic person per year in Singapore),[9] it is imperative that some audit must be done on the management of these patients to ensure more cost-effective management especially because it has long been accepted that evidence-based clinical guidelines for the treatment of asthma (such as GINA guidelines) and asthma education are the best methods available to allow patients to receive a high quality of care and better disease outcomes [10-12]. Although some studies show that the asthma burden may be plateauing,[13-15] this is hypothesized to be caused by an improvement in intervention and prevention efforts. This emphasizes the importance of proper management of asthma to reduce the health care burden of this disease.

With regard to the response rate, the significantly higher response rates in the Philippines and Indonesia were probably because most of the questionnaires were distributed personally to the respondents as compared with the other countries, where the questionnaires were distributed mainly by post. In addition, it must be emphasized that the low response rate for many of the countries may affect the interpretation of the results. Physicians who completed the questionnaire would be assumed to have a greater interest in the management of asthma than the nonresponder group, and thus would probably be more knowledgeable in management of such patients. Thus, the results may reflect an overestimation of the knowledge on this subject of the general population of physicians in Southeast Asia, thus making it safe to conclude that *asthma is treated even less optimally *than is reflected in the results of this study. In addition, as this study was conducted mostly in urban regions, the results may not reflect the practices in the vast rural areas of many of the studied countries. There is a possibility that asthma is treated even more poorly in these areas, as development of programs and resources are limited [16].

Score cards or diaries, peak flow meters and spirometry are used by only a small percentage of physicians to monitor asthma. Except spirometry, the others are relatively inexpensive and convenient methods to monitor asthma. The reasons for these methods being unpopular may be a lack of awareness among physicians about these methods and/or techniques of use, or even perhaps unfamiliarity with the use of these methods to titrate the step-care management of asthma to the child's current level of control. It is suggested that standard scoring systems be implemented for each country, and simple diaries be available in all clinics and hospitals to be given out to all children with asthma seeking treatment. Children should record their symptoms in the diaries and bring them for every follow-up visit thereafter. Cheaply priced peak flow meters should be made available to all practitioners, and perhaps even the patients, so that they may be able to monitor their asthma at home.

The administration of systemic and inhaled corticosteroids in an outpatient setting in the treatment of acute asthma is not favored by some physicians. It has been shown that systemic glucocorticosteroids speed resolution of exacerbations [17,18] and that the combination of high-dose inhaled glucocorticosteroids and salbutamol in acute asthma provides greater bronchodilation than salbutamol alone [19]. The underuse of steroids by physicians in Asia to treat acute asthma has been observed before [20]. Perhaps the use of corticosteroids, whether systemic or inhaled, is not favored by some physicians because of the common misconception that steroids are harmful to young children, particularly with regard to growth, although this theory has long been debunked [21].

A good number of respondents were knowledgeable about the appropriate length of duration for prescription of oral corticosteroids, which was 3 to 5 days, without the need for tailing down of the dose. Regarding use of antibiotics, inappropriate use in acute exacerbations is still present. In western countries, inappropriate antibiotic use is declining, and this is attributed to education of the physicians on appropriate antibiotic use [22,23]. More extensive education of practitioners must be done in the Asia-Pacific to improve this statistic.

For maintenance treatment, it is of concern that many Asian physicians' first choice of therapy in infants and preschoolers is LABA monotherapy. Global Initiative for Asthma guidelines state that LABA should not be used in children 5 years or younger because the safety profile of the drug in that age group has not been sufficiently studied. The LABAs in children older than 5 years should only be used as an add-on therapy for patients whose asthma is insufficiently controlled by medium doses of inhaled glucocorticosteroids or as single-dose therapy before vigorous exercise and not as monotherapy [24]. In fact, there have been studies that show that LABAs may increase the risk of asthma deaths because of the masking of symptoms [25]. It can only be speculated why LABAs have become so popular with physicians, especially in Indonesia, as our data show. Perhaps nonquantifiable factors such as marketing and local practice patterns are at work. We did not analyze the other combinations that physicians indicated as first choice in which LABAs are used with other drugs (such as inhaled corticosteroids). Hence, the statistics of physicians using LABAs at all to treat children younger than 5 years are, in fact, much higher than this study shows.

Inhaled corticosteroids, thankfully, are generally the more popular choice in terms of maintenance therapy for pediatric patients with asthma, with the exception of Indonesia, where the use of LABAs clearly outweigh the use of corticosteroids. It must be reiterated that Global Initiative for Asthma guidelines highly recommend the use of inhaled corticosteroids as maintenance therapy in children even younger than 5 years. It describes inhaled corticosteroids as being the "most effective controller medications currently available."

Of note, montelukast is gaining popularity in Southeast Asia. Montelukast has been studied and shown to be an efficacious therapy for asthma in children aged 2 to 14 years and is generally well tolerated without clinically important adverse effects [26,27]. However, its cost is still relatively high compared with the other forms of therapy, which would greatly influence its prescription because a large percentage of patients would not be able to afford it long-term.

**Figure 1:**
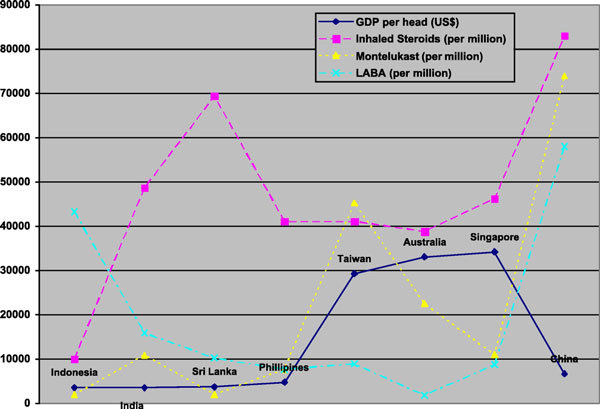
**The GDP per head versus choice of monotherapy in preschoolers (China data may include use as both monotherapy and polytherapy)**.

Certainly, affordability may play a part in the choice of management. A general comparison of choice of therapy in preschoolers versus gross domestic product (GDP) per head [28] demonstrates that more expensive therapy such as montelukast is higher in more affluent nations, whereas cheaper LABA monotherapy is more commonly practiced in areas of lower GDP (Figure 1). Once again, medication pricing and marketing practices are confounders that are not easily ruled out statistically and may very well influence these results. It is encouraging to note the high use of inhaled steroids in most countries regardless of overall affluence.

In conclusion, it must be emphasized that the standard of asthma care in children is likely to be poorer than what this study shows, as mostly urban regions were surveyed. Moreover, the response rate has room for improvement, with responder bias possibly altering the results. Several areas have been identified as having room for improvement, including better monitoring of childhood asthma, and most importantly, less use of LABA monotherapy, especially in young children with asthma.

## Note

APAPARI indicates Asia Pacific Association of Paediatric Allergy, Respirology and Immunology.
